# Correlation of survival and EGFR mutation with predominant histologic subtype according to the new lung adenocarcinoma classification in stage IB patients

**DOI:** 10.1186/1477-7819-12-148

**Published:** 2014-05-19

**Authors:** Yan Sun, Xinmin Yu, Xun Shi, Wei Hong, Jun Zhao, Lei Shi

**Affiliations:** 1 Key Laboratory Diagnosis and Treatment Technology on Thoracic Oncology, 38 Guangji road, Zhejiang province, Hangzhou 310022, People’s Republic of China; 2 Department of Medical Oncology, Zhejiang Cancer Hospital, 38 Guangji road, Gongshu District, Hangzhou 310022, People’s Republic of China

**Keywords:** Lung adenocarcinoma, IASLC/ATS/ERS classification, Stage IB, Prognosis, EGFR mutation

## Abstract

**Background:**

A new lung adenocarcinoma classification proposed by the International Association for the Study of Lung Cancer, American Thoracic Society, and European Respiratory Society (IASLC/ATS/ERS) has recently been published. This study aimed to investigate the utility of the new histological classification for identifying the prognostic subtypes of adenocarcinomas in stage IB patients.Correlations between the classification and the presence of epidermal growth factor receptor (EGFR) mutation status was also studied.

**Methods:**

One hundred and thirty-six patients with stage IB lung adenocarcinoma operated on in Zhejiang Cancer Hospital were identified between 2002 and 2011. Patients overall survival and disease-free survival were calculated using Kaplan-Meier and Cox regression analyses. EGFR mutations were detected using the amplification refractory mutation system.

**Results:**

A total of 136 cases were included in current study, of which 38 were papillary predominant, 39 were acinar predominant, 22 were micropapillary predominant, 21 were lepidic predominant subtypes, 14 were solid predominant, and 2 were variants of invasive adenocarcinoma. Patients with micropapillary- and solid-predominant tumors had the lowest five-year disease-free survival (28.4 and 36.7%, respectively). Univariate and multivariate analysis showed that the micropapillary-predominant subtype was an independent predictor of disease-free survival (*P* = 0.0041 and 0.048, respectively), but not overall survival (*P* = 0.175 and 0.214, respectively). EGFR mutations were significantly associated with the micropapillary-predominant subtype patients (*P* = 0.0026). The EGFR mutation frequency is lower in the solid-predominant subtype than other subtypes (*P* = 0.0508).

**Conclusions:**

The predominant subtype in the primary tumor was associated with prognosis in resected stage IB lung adenocarcinoma. The EGFR mutation frequency of micropapillary-predominant subtype is higher than other subtypes.

## Background

Lung cancer is theleading cause of death in cancer patients worldwide [1]. The incidences of stage I non-small cell lung cancer (NSCLC) has increased recent years. However, recurrence and metastasis is a great challenge in these patients. The five-year survival rates of patients with resected stage I NSCLC is about 60% [2]. There is a great need to predict which patients will recur in order to develop strategies for choosing which patients may benefit by adjuvant treatment. According to the 2013 National Comprehensive Cancer Network Clinical Practice Guidelines in Oncology for NSCLC, stage pathologic tumor node metastasis (pTNM) IB (tumor size more than 3 cm and less than 5 cm or pleural involvement) patients with complete resection are treated with observation except for the patients with high risk factors such as lymphatic and/or vessel invasion, pleural involvement, and a tumor size of more than 4 cm. However, there is some dispute in this setting.

A new lung adenocarcinoma classification proposed by the International Association for the Study of Lung Cancer, American Thoracic Society, and European Respiratory Society (IASLC/ATS/ERS) has recently been published [3]. Many researchers have attempted to implement a more meaningful pathological classification which could provide prognostic and molecular biology information with relevance to clinical behavior [4-7].

Epidermal growth factor receptor (EGFR) mutations have been recently discovered and EGFR- -tyrosine kinase inhibitor (TKI treatment has been played an important role in treating advanced NSCLC, especially for EGFR mutation patients [8-10]. The frequency of EGFR mutation has proven to be more common in adenocarcinoma [11]. However, the relationship between the EGFR mutation and the subtype of the new lung adenocarcinoma classification is not clear.

This study reviewed a series of consecutive patients with stage IB NSCLC who had been operated on in a single institution according to the new IASLC/ATS/ERS classification.It tracked the relationship between the predominant subtype of adenocarcinoma and prognosis, in addition to detecting the Correlation between the new subtype ofadenocarcinoma and EGFR mutations.

## Methods

### Patient eligibility

A total of 136 adenocarcinoma patients with pathologic stage IB NSCLC, who underwent complete resection between January 2002 and December 2011, were identified in Zhejiang Cancer Hospital. The Ethics Committee at Zhejiang Cancer Hospital approved the study. All patients underwent complete resection of lung cancer. Histological typing was determined as adenocarcinoma according to the 2004 World Health Organization classification. Lung cancer staging was performed for all patients according to the seventh tumor node metastasis (TNM) staging classification. All of the patients did not receive preoperative chemotherapy or radiation therapy.

### Histological evaluation

All resected specimens were formalin fixed and stained with hematoxylin and eosin in the routine manner. Each of the slides was examined independently by three specialists. The average number of slides from each case reviewed in our study was 11.5 (range: 1 to 35). Histological classification was according to the IASLC/ATS/ERS classification of lung adenocarcinoma and the 2004 WHO classification. According to the IASLC/ATS/ERS criteria, each tumor was reviewed using comprehensive histologic subtyping, recording the percentage in 5% increments for each histologic component. Tumors were classified as adenocarcinomas *in situ* (AIS), minimally invasive adenocarcinomas (MIA), and invasive adenocarcinomas. Adenocarcinomas were further subdivided into lepidicpredominant (Lepidic), papillarypredominant (Pap), acinarpredominant (Aci), micropapillarypredominant (MP), solidpredominant (Solid), invasive mucinous adenocarcinoma and others (including colloid adenocarcinoma and fetal adenocarcinoma). The predominant pattern is defined as the pattern with the largest percentage.

### EGFR mutation analysis

Molecular analysis of EGFR was performed using the amplification refractory mutation system (ARMS) with formalin-fixed paraffin embedded archival tissue blocks obtained during surgical excision of the tumors. The examination method followed was that of Lynch *et al*. [12].

### Follow-up

Patients were examined at three-month intervals for the first two years and at six-month intervals thereafter. The follow-up evaluation included a physical examination, a computed tomography scan of the chest and abdomen, brain magnetic resonance imaging, and bone scintigraphy. The last follow-up date was 31 May 2013. The median survival time from surgery to the last censoring date was 74 months (range, 21 to 145 months).

## Results

### Clinicopathologic and histologic features

The clinicopathologic characteristics of the patients are listed in Table 1. The median age of the patients was 57.6 years in the current cohort. Of the136 patients, 103 patients were never smokers and 33 were former or current smokers.

**Table 1:** Demographic characteristics of the study population

	**Number of patients**
Gender	
Male	79
Female	57
Age (years)	
Range	34-79
Median	57.6
<65	95
≥65	41
Smoking status	
Never	103
Former or current	33
Tumor size	
≥3 cm	89
<3 cm	47
Adjuvant chemotherapy	
Yes	37
No	99
Grade	
Well	57
Moderate or poor	79
Surgical procedure	
Lobectomy	119
Bilobectomy	17
Pleural involvement	
Yes	69
No	67
Lymphatic and/or vessel invasion	
Yes	12
No	124
Histological subtypes (IASLC/ATS/ERS)	
Invasive adenocarcinoma	
Lepidicpredominant	21
Acinarpredominant	39
Papillarypredominant	38
Micropapillarypredominant	22
Solid-predominant	14
Variants of invasive adenocarcinoma	2

Of the 136 invasive adenocarcinoma cases, 38 were papillarypredominant, 39 were acinarpredominant, 22 were micropapillarypredominant, 14 were solidpredominant, 21 were lepidicpredominant subtypes, and 2 were variants of invasive adenocarcinoma (Table 1). A total of 12 specimens showed a single pattern, whereas 32 displayed two, 54 displayed three, and 40 displayed at least four patterns. Two patients were variants of invasive adenocarcinoma (colloid adenocarcinoma).

### Survival analyses

**Figure 1:**
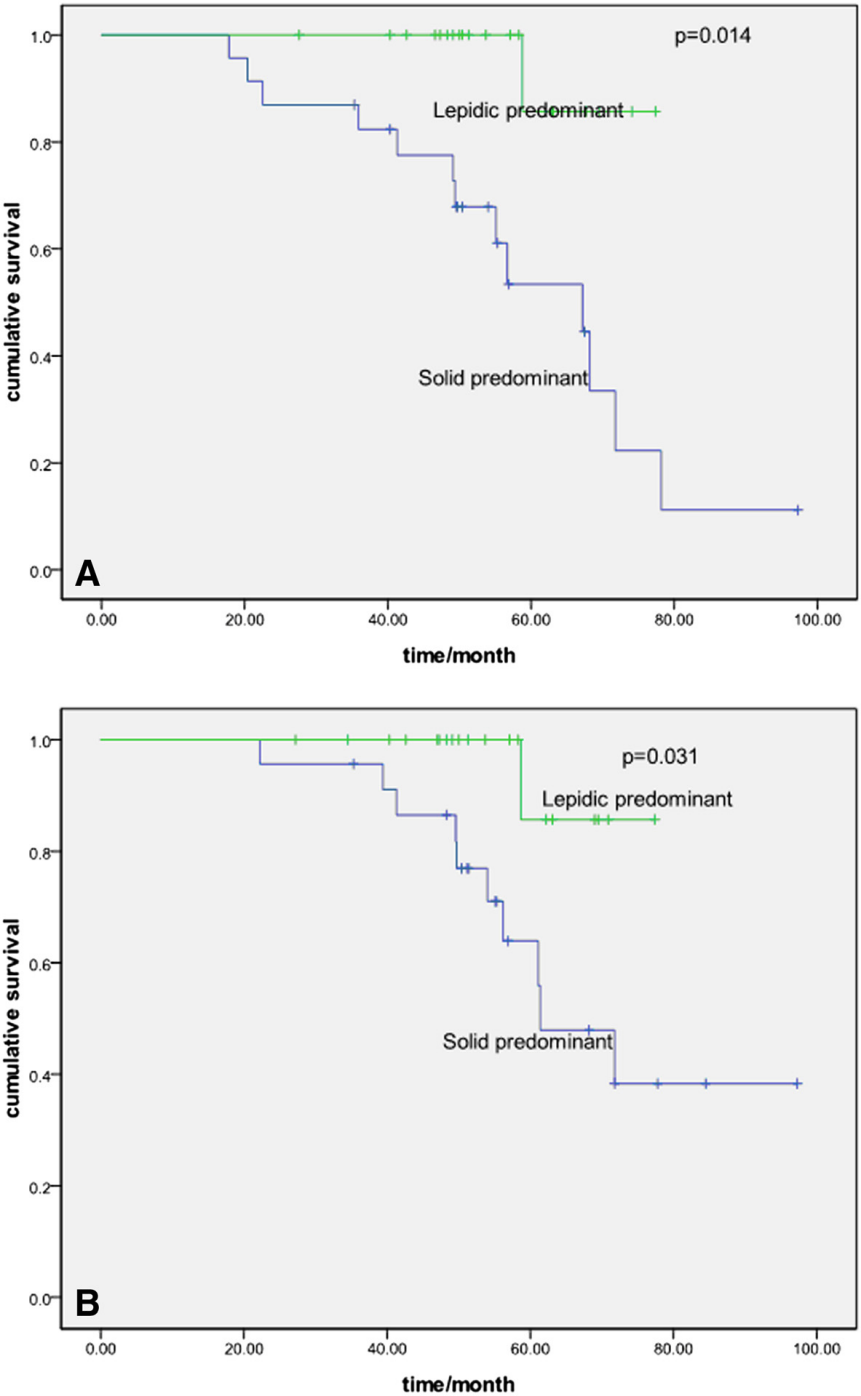
**Probability of disease-free survival and overall survival between lepidic-and solid-predominant subtype. A** Probability of disease-free survival comparison between lepidic-and solid-predominant subtype (*P* = 0.014)**. B** Probability of overall survival comparison between lepidic- and solid-predominant subtype (*P* = 0.031).

**Figure 2:**
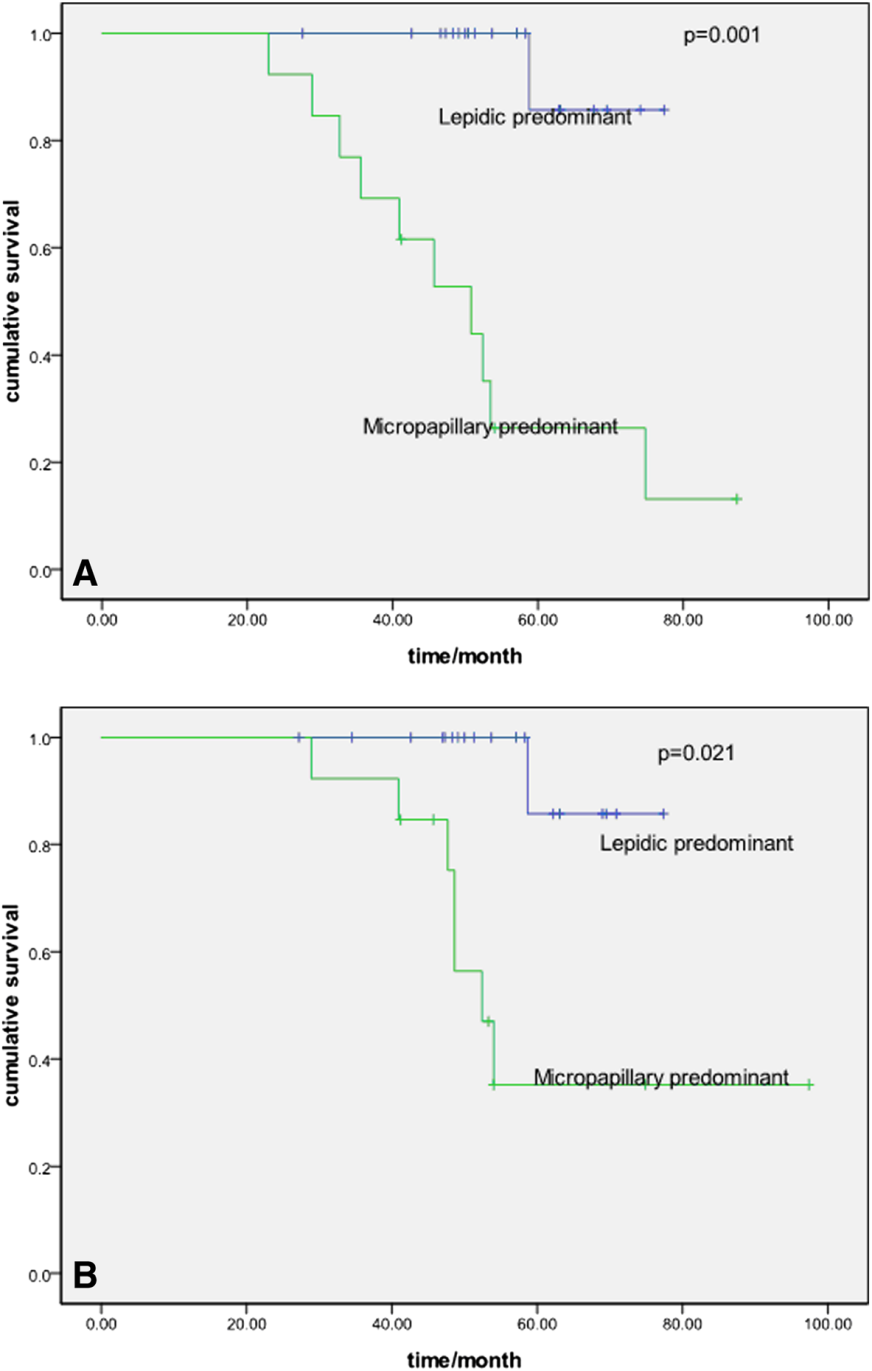
**Probability of disease-free survival and overall survival between lepidic- and micropapillary-predominant subtype. A** Probability of disease-free survival comparison between lepidic- and micropapillary-predominant subtype (*P* = 0.001)**. B** Probability of overall survival comparison between lepidic- and micropapillary-predominant subtype (*P* = 0.021).

**Figure 3:**
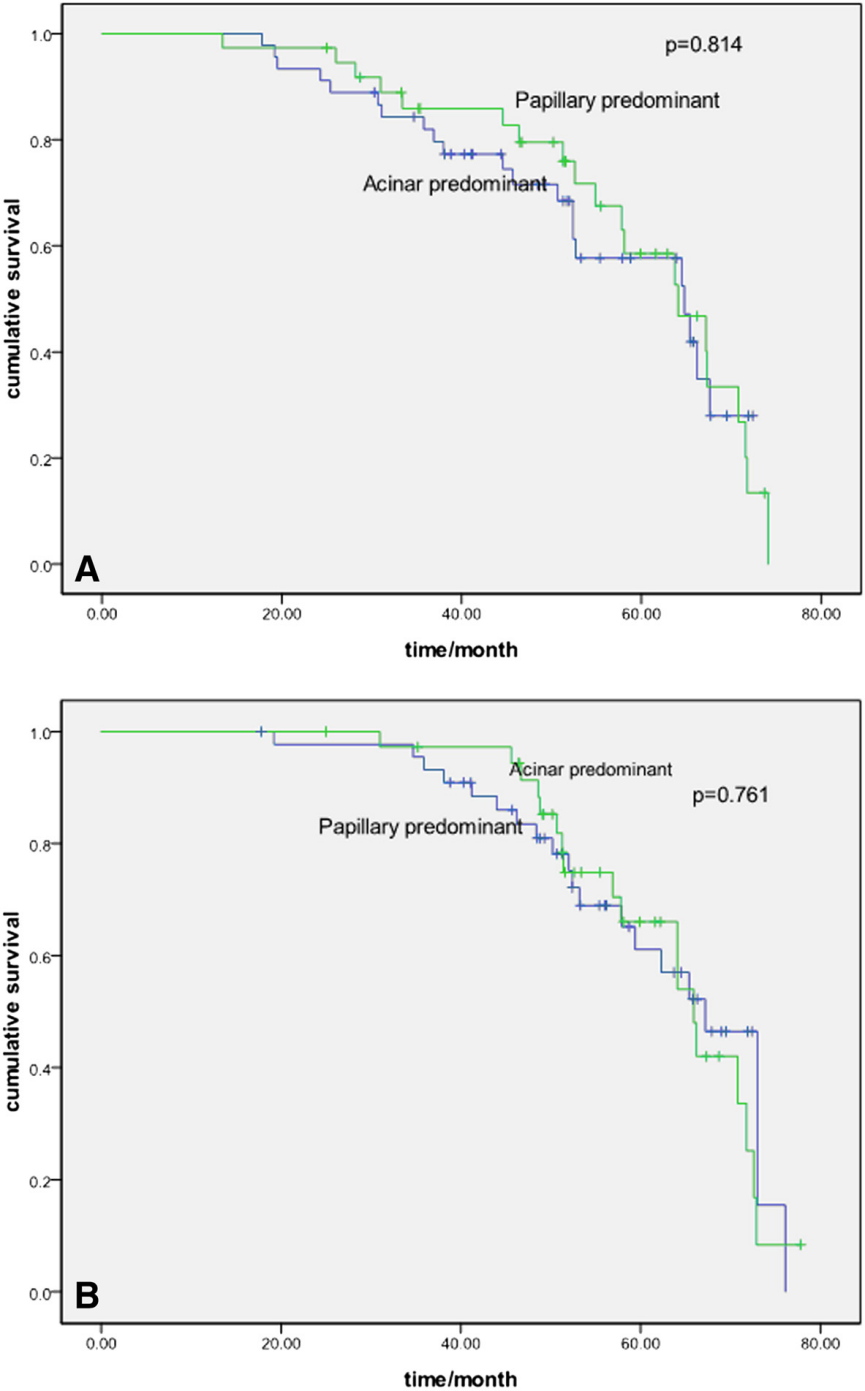
**Probability of disease-free survival and overall survival between acinar- and papillary-predominant subtype. A** Probability of disease-free survival comparison between acinar- and papillary-predominant subtype (*P* = 0.814)**. B** Probability of overall survival comparison between acinar- and papillary-predominant subtype (*P* = 0.761).

Table 2 shows the results of the univariate analyses of the clinicopathologic and histologic factors evaluated in this study. The five-year disease-free survival rate (DFS) and overall survival rate (OS) for the all patients were 52.4 and 62.0%, respectively. Lymphatic and/or vessel invasion, micropapillary-predominant subtype, and solid-predominant subtype correlated significantly with a worse DFS (*P* = 0.045, *P* = 0.041, and *P* = 0.049, respectively), although these were not significantly different for OS. There was a significant difference between different histologic subtypes for DFS but not for OS (Figures 1, 2 and 3).

**Table 2:** Univariate analysis of patient survival according to clinicopathologic characteristics

	**5-year DFS rate (%)**	***P***	**5-year OS rate (%)**	***P***
Gender		0.313		0.601
Male (n = 79)	53.4		60.7	
Female (n = 57)	51.7		66.7	
Age (years)		0.643		0.475
<65 (n = 95)	50.1		59.7	
≥65 (n = 41)	56.8		67.2	
Smoking status		0.541		0.312
Never (n = 103)	56.5		70.6	
Former or current (n = 33)	46.5		54.5	
Tumor size		0.214		0.295
>3 cm (n = 89)	46.7		59.7	
≤3 cm (n = 43)	54.2		66.2	
Adjuvant chemotherapy		0.213		0.713
Yes (n = 37)	59.1		67.9	
No (n = 99)	49.0		60.3	
Grade		0.675		0.233
Well (n = 57)	59.6		73.3	
Moderate or poor (n = 79)	50.1		55.4	
Pleural involvement		0.769		0.961
Yes (n = 69)	49.0		60.4	
No (n = 67)	55.2		67.5	
Lymphatic and/or vessel invasion		0.045		0.112
Yes (n = 12)	39.1		51.7	
No (n = 124)	54.3		68.1	
Histological subtypes (IASLC/ATS/ERS)				
Lepidicpredominant		0.042		0.307
Yes (n = 21)	75.2		80.8	
No (n = 115)	50.8		59.3	
Acinarpredominant		0.782		0.443
Yes (n = 39)	55..5		67.8	
No (n = 97)	49.0		58.9	
Papillary predominant		0.401		0.405
Yes (n = 38)	57.6		72.1	
No (n = 98)	47.1		57.2	
Micropapillary predominant		0.041		0.175
Yes (n = 22)	28.4		43.5	
No (n = 114)	61.1.		62.9	
Solid predominant		0.049		0.211
Yes (n = 14)	36.7		45.9	
No (n = 122)	57.7		65.1	
Variants of invasive adenocarcinoma		0.315		0.306
Yes (n = 2)	100		100	
No (n = 134)	51.3		60.5	

DFS, disease-free survival rate; OS, overall survival rate.

A multivariate Cox’s regression model was constructed considering lymphatic and/or vessel invasion, micropapillary predominant, solid predominant, and lepidic predominant as variables. Micropapillary predominant qualified as an independent prognostic factor for DFS but not OS (Table 3).

**Table 3:** Multivariate survival analysis for disease-free survival and overall survival

		**DFS**		**OS**		
	**HR**	**95**% **CI**	***P***	**HR**	**95**% **CI**	***P***
Lepidic predominant (yes/no)	0.756	0.723-1.012	0.101	0.876	0.597-1.986	0.742
Micropapillary predominant (yes/no)	1.473	1.041-1.786	0.048	1.342	0.764-1.987	0.214
Solid predominant (yes/no)	1.210	0.987-1.978	0.076	1.229	0.746-2.158	0.435
lymphatic and/or vessel invasion (yes/no)	1.153	0.979-2.143	0.079	1.075	0.774-3.547	0.843

DFS, disease-free survival; OS, overall survival.

### Correlation with EGFR mutation and clinicopathologic characteristics

A total of 102 out of 136 patients provided tumor samples for EGFR mutation analysis. EGFR mutations were identified in 39 (38.2%) patients (22 with exon 19 delete and 17 with exon 21 L858R mutation). There was a significant difference among the patients with EGFR mutation frequency between the micropapillary-predominant subtype and other subtypes (*P* = 0.0026). In contrast, EGFR mutations were less frequent in the solid-predominant subtype than in the other subtypes (*P* = 0.0508) (Table 4). There was no significant difference between other subtypes and EGFR mutations.

**Table 4:** Predominant histologic subtypes and their correlation with epidermal growth factor receptor mutation (EGFR) in 102 patients

	**EGFR mutation number (n = 39)**	**Wild-type number (n = 63)**	***P***
Lepidic predominant			0.29
Yes	8 (20.5%)	8 (12.7%)	
No	31 (79.5%)	55 (87.3%)	
Acinar predominant			0.27
Yes	9 (23.1%)	21 (33.3%)	
No	30 (76.9%)	42 (66.7%)	
Papillary predominant			0.57
Yes	8 (20.5%)	16 (25.4%)	
No	31 (79.5%)	47 (74.6%)	
Micropapillary predominant			0.0026
Yes	12 (30.7%)	5 (7.9%)	
No	27 (69.3%)	58 (92.1%)	
Solid predominant			0.0508
Yes	1 (2.6%)	11 (17.5%)	
No	38 (97.4%)	52 (82.5%)	
Variants of invasive adenocarcinoma			0.69
Yes	0 (0.0%)	2 (3.2%)	
No	39 (100%)	61 (96.8%)	

## Discussion

In the presented study, the data indicated that the IASLC/ATS/ERS histologic subtypes of lung adenocarcinoma could predict the prognosis of stage IB patients. EGFR-mutated tumors were more likely to be of the micropapillary-predominant subtype and were less frequent in the solid-predominant subtype. The prognostic impact of the new classification on recurrence has been validated in several studies [13,14]. Yoshizawa *et al*. [7] reported that the IASLC/ATS/ERS histologic classification was predictive of prognosis in stage I adenocarcinoma. Their data revealed that AIS and MIA had a 100.0% five-year DFS. Lepidic predominant, papillary predominant, and acinar-predominant subtypes had a 90.0%, 83.0%, and 84.0% five-year DFS, respectively. In the study by Hung *et al*. [14], the lepidic-predominant adenocarcinomas had a lower risk of recurrence, whereas micropapillary- and solid-predominant adenocarcinomas had a higher risk for recurrence. In our study, the lepidic-predominant adenocarcinomas were shown to have a better prognosis compared with other subtypes.The micropapillary- and solid-predominant adenocarcinomas had the worst prognosis, which is similar to the results from the Hung *et al.* study [14].

The prognostic value of the new classification on OS has also been researched in several studies [4-7,13,14]. Micropapillary- and solid-predominant adenocarcinomas showed poor OSwhen compared with other subtypes in the Song *et al.* study [15]. However, no survival difference for post-recurrence was identified among different subtypes in the Hung *et al.* cohort study [14]. In our cohort study, no difference was found in thefive-year OS between different histology subtypes in univariate and multivariate analyses, which may due to the different treatment after recurrence or metastases, such as EGFR-TKI therapy.

The relationship between EGFR mutations and predominant subtype has been examined in several studies [16,17]. The data between the EGFR mutation and histology subtype are conflicting. Zhang *et al. *[17] investigated 349 lung adenocarcinoma cases and found EGFR mutations were more frequent in acinar-predominant tumors. However, EGFR mutations were found to be more frequent in micropapillary-predominant tumors in the Shim *et al.* and Song *et al.* studies [16,18]. Our results showed that EGFR mutations was more frequent in micropapillary-predominant subtypes (*P* = 0.0026). The different outcome between EGFR mutations and histology subtypes may be related to study sample size and ethnic difference.

Our study’s major limitations were being retrospective and from a single institution. In addition, EGFR mutation data was not available for all of the patients, thereby limiting the inferences possible from our clinical study. However, with the small number of patients in clinical trials, our retrospective study is still meaningful.

## Conclusions

In conclusion, we have demonstrated the prognostic value of the new classification in stage IB lung adenocarcinoma patients. This new classification might be valuable for detecting patients with a high risk of recurrence in order for them to receive postoperative adjuvant treatment. EGFR mutations were found more frequently in micropapillary-predominant tumors in this study.

## Abbreviations

EGFR: epidermal growth factor receptor; TKI: tyrosine kinase inhibitor; TNM: tumor node metastasis.

## Competing interests

The authors declare that they have no competing interests.

## Authors’ contributions

YS and XY cooperated in the conception and design of the study, and in the collection of the data; JZ,XS and WH validated all pathology reports, and assisted in data analysis and interpretation of data; YS drafted the manuscript. All authors approved the final manuscript.

## References

[B1] Siegel R Naishadham D Jemal A Cancer statistics, 2013 CA Cancer J Clin 2013 63 11 30 10.3322/caac.21166 23335087

[B2] Li Z Yu Y Lu J Luo Q Wu C Liao M Zheng Y Ai X Gu L Lu S Analysis of the T descriptors and other prognosis factors in pathologic stage I non-small cell lung cancer in China J ThoracOncol 2009 4 702 709 10.1097/JTO.0b013e3181a5269d 19404215

[B3] Travis WD Brambilla E Noguchi M Nicholson AG Geisinger KR Yatabe Y Beer DG Powell CA Riely GJ Van Schil PE Garg K Austin JH Asamura H Rusch VW Hirsch FR Scagliotti G Mitsudomi T Huber RM Ishikawa Y Jett J Sanchez-Cespedes M Sculier JP Takahashi T Tsuboi M Vansteenkiste J Wistuba I Yang PC Aberle D Brambilla C Flieder D The new IASLC/ATS/ERS international multidisciplinary lung adenocarcinoma classification J Thoracic Oncol 2011 6 244 285 10.1097/JTO.0b013e318206a221 PMC4513953 21252716

[B4] Woo T Okudela K Mitsui H Tajiri M Yamamoto T Rino Y Ohashi K Masuda M Prognostic value of the IASLC/ATS/ERS classification of lung adenocarcinoma in stage I disease of Japanese cases PatholInt 2012 62 785 791 10.1111/pin.12016 23252867

[B5] Gu J Lu C Guo J Chen L Chu Y Ji Y Ge D Prognostic significance of the IASLC/ATS/ERS classification in Chinese patients-A single institution retrospective study of 292 lung adenocarcinoma J SurgOncol 2013 107 474 480 10.1002/jso.23259 22952152

[B6] Russell PA Barnett SA Walkiewicz M Wainer Z Conron M Wright GM Gooi J Knight S Wynne R Liew D John T Correlation of mutation status and survival with predominant histologic subtype according to the new IASLC/ATS/ERS lung adenocarcinoma classification in stage III (N2) patients J ThoracOncol 2013 8 461 468 10.1097/JTO.0b013e3182828fb8 23486266

[B7] Yoshizawa A Motoi N Riely GJ Sima CS Gerald WL Kris MG Park BJ Rusch VW Travis WD Impact of proposed IASLC/ATS/ERS classification of lung adenocarcinoma: prognostic subgroups and implications for further revision of staging based on analysis of 514 stage I cases Mod Pathol 2011 24 653 664 10.1038/modpathol.2010.232 21252858

[B8] Mitsudomi T Morita S Yatabe Y Negoro S Okamoto I Tsurutani J Seto T Satouchi M Tada H Hirashima T Asami K Katakami N Takada M Yoshioka H Shibata K Kudoh S Shimizu E Saito H Toyooka S Nakagawa K Fukuoka M Gefitinib versus cisplatin plus docetaxel in patients with non-small-cell lung cancer harbouring mutations of the epidermal growth factor receptor (WJTOG3405): an open label, randomised phase 3 trial Lancet Oncol 2010 11 121 128 10.1016/S1470-2045(09)70364-X 20022809

[B9] Maemondo M Inoue A Kobayashi K Sugawara S Oizumi S Isobe H Gemma A Harada M Yoshizawa H Kinoshita I Fujita Y Okinaga S Hirano H Yoshimori K Harada T Ogura T Ando M Miyazawa H Tanaka T Saijo Y Hagiwara K Morita S Nukiwa T Gefitinib or chemotherapy for non-small-cell lung cancer with mutated EGFR N Engl J Med 2010 362 2380 2388 10.1056/NEJMoa0909530 20573926

[B10] Shepherd FA Rodrigues Pereira J Ciuleanu T Tan EH Hirsh V Thongprasert S Campos D Maoleekoonpiroj S Smylie M Martins R van Kooten M Dediu M Findlay B Tu D Johnston D Bezjak A Clark G Santabárbara P Seymour L Erlotinib in previously treated non-small-cell lung cancer N Engl J Med 2005 353 123 132 10.1056/NEJMoa050753 16014882

[B11] Mok TS Wu YL Thongprasert S Yang CH Chu DT Saijo N Sunpaweravong P Han B Margono B Ichinose Y Nishiwaki Y Ohe Y Yang JJ Chewaskulyong B Jiang H Duffield EL Watkins CL Armour AA Fukuoka M Gefitinib or carboplatin-paclitaxel in pulmonary adenocarcinoma N Engl J Med 2009 361 947 957 10.1056/NEJMoa0810699 19692680

[B12] Lynch TJ Bell DW Sordella R Gurubhagavatula S Okimoto RA Brannigan BW Harris PL Haserlat SM Supko JG Haluska FG Louis DN Christiani DC Settleman J Haber DA Activating mutations in the epidermal growth factor receptor underlying responsiveness of non-small-cell lung cancer to gefitinib N Engl J Med 2004 350 2129 2139 10.1056/NEJMoa040938 15118073

[B13] Yoshizawa A Sumiyoshi S Sonobe M Kobayashi M Fujimoto M Kawakami F Tsuruyama T Travis WD Date H Haga H Validation of the IASLC/ATS/ERS lung adenocarcinoma classification for prognosis and association with EGFR and KRAS gene mutations: analysis of 440 Japanese patients J ThoracOncol 2013 8 52 61 10.1097/JTO.0b013e3182769aa8 23242438

[B14] Hung JJ Jeng WJ Chou TY Hsu WH Wu KJ Huang BS Wu YC Prognostic value of the New international association for the study of lung cancer/American thoracic society/European respiratory society lung adenocarcinoma classification on death and recurrence in completely resected stage I lung adenocarcinoma Ann Surg 2013 258 1079 1086 10.1097/SLA.0b013e31828920c0 23532112

[B15] Song Z Zhu H Guo Z Sun W Zhang Y Prognostic value of the IASLC/ATS/ERS classification in stage I lung adenocarcinoma patients-based on a hospital study in China Eur J SurgOncol 2013 39 1262 1268 10.1016/j.ejso.2013.08.026 24063970

[B16] Shim HS da Lee H Park EJ Kim SH Histopathologic characteristics of lung adenocarcinomas with epidermal growth factor receptor mutations in the international association for the study of lung cancer/American thoracic society/European respiratory society lung adenocarcinoma classification Arch Pathol Lab Med 2011 135 1329 1334 10.5858/arpa.2010-0493-OA 21970488

[B17] Zhang Y Sun Y Pan Y Li C Shen L Li Y Luo X Ye T Wang R Hu H Li H Wang L Pao W Chen H Frequency of driver mutations in lung adenocarcinoma from female never-smokers varies with histologic subtypes and age at diagnosis Clin Cancer Res 2012 18 1947 1953 10.1158/1078-0432.CCR-11-2511 22317764 PMC3319848

[B18] Song Z Zhu H Wu W Sun W Zhang Y Correlation of EGFR mutation and predominant histologic subtype according to the new lung adenocarcinoma classification in Chinese patients Med Oncol 2013 30 645 23797772 10.1007/s12032-013-0645-1

